# Monochromic Radiations Provided by Light Emitted Diode (LED) Modulate Infection and Defense Response to Fire Blight in Pear Trees

**DOI:** 10.3390/plants10091886

**Published:** 2021-09-12

**Authors:** Tiziana Sgamma, Ivano Forgione, Francesca Luziatelli, Calogero Iacona, Roberto Mancinelli, Brian Thomas, Maurizio Ruzzi, Rosario Muleo

**Affiliations:** 1 Biomolecular Technology Group, Leicester School of Allied Health Science, De Montfort University, Leicester LE1 9BH, UK; tiziana.sgamma@dmu.ac.uk; 2 Department of Agricultural and Forestry Sciences, Tuscia University, Via S. C. De Lellis, snc., 01100 Viterbo, Italy; ivano.forgione@unitus.it (I.F.); mancinel@unitus.it (R.M.); 3 Department for Innovation in Biological, Agro-Food and Forest Systems (DIBAF), Tuscia University, Via S. C. De Lellis, snc., 01100 Viterbo, Italy; f.luziatelli@unitus.it (F.L.); ruzzi@unitus.it (M.R.); 4 Department of Agriculture, Food and Environment (DAFE), University of Pisa, Via del Borghetto, 80, 56124 Pisa, Italy; calogero.iacona@unipi.it; 5 School of Life Sciences, University of Warwick, Coventry CV4 7AL, UK; Brian.Thomas@warwick.ac.uk

**Keywords:** light quality, photosensors, host-pathogen interaction, resistance genes, gene regulation, bacterial growth, *Erwinia amylovora*, circadian rhythms, optogenetics

## Abstract

Pathogenesis-related (PR) proteins are part of the systemic signaling network that perceives pathogens and activates defenses in the plant. Eukaryotic and bacterial species have a 24-h ‘body clock’ known as the circadian rhythm. This rhythm regulates an organism’s life, modulating the activity of the phytochromes (*phys*) and cryptochromes (*crys*) and the accumulation of the corresponding mRNAs, which results in the synchronization of the internal clock and works as zeitgeber molecules. Salicylic acid accumulation is also under light control and upregulates the *PR* genes expression, increasing plants’ resistance to pathogens. *Erwinia amylovora* causes fire blight disease in pear trees. In this work, four bacterial transcripts (*erw1-4*), expressed in asymptomatic *E. amylovora*-infected pear plantlets, were isolated. The research aimed to understand how the circadian clock, light quality, and related photoreceptors regulate *PR* and *erw* genes expression using transgenic pear lines overexpressing *PHYB* and *CRY1* as a model system. Plantlets were exposed to different circadian conditions, and continuous monochromic radiations (Blue, Red, and Far-Red) were provided by light-emitting diodes (LED). Results showed a circadian oscillation of *PR10* gene expression, while *PR1* was expressed without clear evidence of circadian regulation. Bacterial growth was regulated by monochromatic light: the growth of bacteria exposed to Far-Red did not differ from that detected in darkness; instead, it was mildly stimulated under Red, while it was significantly inhibited under Blue. In this regulatory framework, the active form of phytochrome enhances the expression of *PR1* five to 15 fold. An ultradian rhythm was observed fitting the zeitgeber role played by CRY1. These results also highlight a regulating role of photoreceptors on the expression of *PR*s genes in non-infected and infected plantlets, which influenced the expression of *erw* genes. Data are discussed concerning the regulatory role of photoreceptors during photoperiod and pathogen attacks.

## 1. Introduction

*Erwinia amylovora* causes fire blight, a disease of agronomic and economic importance that affects many Rosaceae species, primarily pear and apple trees. Bacteria penetrate the plant mainly through the flowers and can also enter leaf tissue through wounds [1]. During plant-pathogen interaction, a dialogue occurs between the two organisms: the plant synthesizes molecules for the signaling system and defense; in contrast, the pathogen synthesizes molecules to break down the barriers of the host and mimic plant hormones. In the beginning, under favorable climatic conditions, the pathogen inserts through the intercellular spaces of parenchyma. Afterward, it colonizes the xylem vessels causing extensive damage with the final death of the plant [2]. The necrotic parts of the plant become brown as if they had been burned by fire [3].

During the infection process, pathogenesis-related (PR) proteins are part of an articulated systemic signaling network active in plants to perceive pathogens and activate defenses. The necrotic lesion induces the expression of a set of pathogenesis-related genes. *PR1* gene, one of the 17 *PR* gene families, has frequently been used as a marker for systemic acquired resistance (SAR) in many plant species [4,5,6]. In the immunization stage, necrosis-causing pathogen when infects a leaf usually provokes the formation of a localized dry, necrotic lesion that limits pathogen spread and provides local resistance. This step is also referred to as hypersensitive response (HR) [7]. Accumulation of salicylic acid (SA) is also associated with this stage. SA is an endogenous plant hormone whose levels increase after pathogen infection. SA can induce the expression of *PR1* [8]. Increases in SA and SA-inducible PR proteins are associated with disease resistance at several levels, not just with the SAR response. A phloem-mobile signal then moves from the immunized leaf to the rest of the plant to establish SAR. The perception of the mobile signal in the uninoculated leaves results in the expression of the same set of *PR* genes as induced around the primary infection site. When the plant is challenged with a second virulent pathogen, the plant responds as if that was an avirulent one because of the rapid accumulation of the *PR1*-transcripts [9]. However, there are indications that several of the *PR* genes are expressed at basal levels in plants without any pathogen attack. Moreover, studies showed that SA is also crucial for sustaining basal levels of genes associated with resistance responses, including *PR1*, and keeping the defense system primed in the absence of pathogen attacks [4,10,11,12,13]. Remarkably, when *PR* genes are not expressed this leads to a higher susceptibility to infectious agents [5].

The PR10s defense-related proteins are a ubiquitous class of intracellular in contrast to the extracellular nature of most PR proteins [14]. Most of them are induced upon microbial attack by fungal elicitors, wounding, and stress stimuli, as with most of the other PR-protein families. PR10 proteins are also expressed in a tissue-specific manner during development and some PR10 proteins show constitutive expression patterns [15,16]. PR10s have been attributed a ribonuclease-like function due to sequence homology with ribonucleases (RNase) [17]. However, only some PR10 proteins have been proposed to possess RNase activity [18]. In addition, they have also been shown to respond to plant hormones, including jasmonic acid (JA), and abiotic stresses such as salt and drought [18].

Plant defense responses at the site of the bacterial infection are elevated, accumulation of SA occurs and the transcription of *PR1* is induced in light [19]. The PR1 light dependency and the execution of HR confirm that these responses are closely associated and that light regulation already takes place early in this SA-dependent signaling pathway [7]. Phytochromes are crucial photoreceptors and are involved in the modulation of the *PR1* expression by light. The absence of both PHYA and PHYB strongly reduces the expression of *PR* genes upon treatment with SA, with a more significant influence of PHYB deficiency [5,7,20]. Phytochrome signaling strongly modulates the response of endogenous SA [5]. There is a strict light dependency of gene expression of *PRs* and the HR process. HR lesions are often correlated with the induction of *PR* genes and are also light modulated. HR is strongly reduced by the absence of phytochromes and amplified in an SA-dependent manner in the *psi2* mutant [5].

Moreover, photoreceptor proteins such as cryptochromes (CRYs), which are Blue light (BL) photoreceptors homologous to photolyases, seem to be involved in pathogen response. Proteomics study identified proteins with altered expression related to defense, stress, and detoxification in *cry1* mutant [21]. CRY1 positively regulates SAR, indeed, in *Arabidopsis*, the inactivation of the *CRY1* gene has a mild influence on the SA accumulation and determines a reduction of the *PR1* expression; in contrast, the overexpression of this gene *CRY1* significantly enhances the expression of *PR1* [22]. Furthermore, other studies showed that in mutants of COP1 (constitutive photomorphogenesis 1), COP9, and DET1 (De-etiolated 1), which are part of the CRY1 signaling pathway, *PR* genes were highly up-regulated [23,24].

Prokaryotes have evolved a repertoire of photosensory proteins that determine changes in the external light and regulate cell physiology in a light-dependent manner [25]. Bacterial photoreceptors include proteins with a bilin-type chromophore (bacteriophytochromes) for sensing red light (RL) and far-red light (FRL) [26]. Moreover, they include proteins with photosensory domains for BL such as BLUF (BL sensing using flavin adenine dinucleotide [FAD]), LOV (light, oxygen, or voltage), PYP (photoactive yellow protein), and cryptochrome/photolyase (Cry/PHR) superfamilies, green- or blue-light-absorbing microbial rhodopsin [27,28]. In plant-associated bacteria, the number of candidate photoreceptors varies: *Pseudomonas syringae* pv *syringae* B728a and *Pseudomonas syringae* pv *tomato* DC3000 have two bacteriophytochromes (BphP1 and BphP2) and one LOV domain-containing histidine kinase (LOV-HK) [29]; anoxygenic phototrophs, such as Methylobacteria, can contain between 3 and 16 photosensory proteins [30].

In the Enterobacteriaceae, there is only one report indicating the presence of a *bphP* gene in *Enterobacter cloacae* [31]. At the same time, several studies show that these bacteria are sensitive to irradiation treatments with wavelengths in the range of visible, violet, and blue light [32,33,34]. So far, no gene encoding photosensory protein has been identified yet in the plant pathogenic Enterobacteriaceae species *E. amylovora*, and there is no evidence that the growth, phenotype, or virulence of this pathogen is affected by the light.

In this work, four *E. amylovora* genes (*erw*) were isolated that could be used as a marker to monitor the initial phase of the infection in asymptomatic plants. To investigate if the circadian internal clock, the light quality, and the related photoreceptors autonomously regulate the abundance of *PR1* and *PR10* transcripts, in vitro-cultured plantlets of Iranian pear cultivar *Dar Gazi-wild type* (*wt*), *Dar Gazi-phyB* (transgenic plant overexpressing *Arabidopsis phyB*, and *Dar Gazi-cry1* (transgenic plant overexpressing tomato *CRY1*) were exposed to different circadian experimental conditions, and continuous BL, RL, and FRL, emitted by light-emitting diodes (LED).

Transcriptional changes in host and pathogen gene expression during early *E. amylovora* infection indicated that both plant *PR*s and bacterial *erw* genes were temporarily expressed and differentially regulated. The results reported in this work indicate that photoreceptor-mediated signals regulate the expression of specific plant and pathogen genes in pear plantlets infected by *E. amylovora*.

## 2. Results

### 2.1. Effect on the Monochromatic Light on the Growth of E. amylovora In Vitro

**Figure 1:**
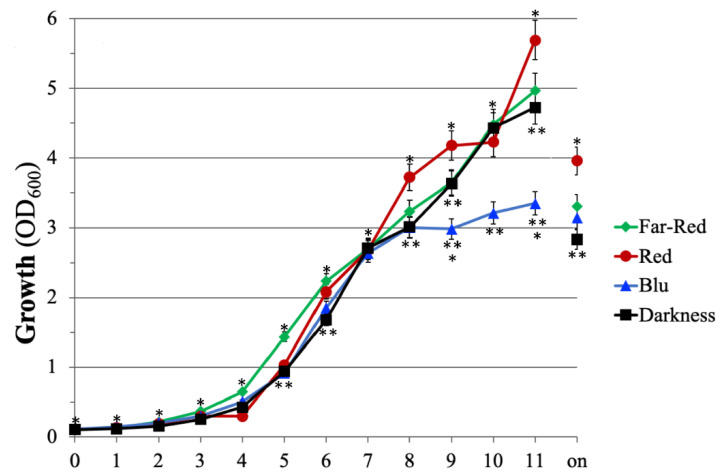
The effect of monochromatic light on the growth of *E. amylovora* Ea273 in shake flask cultures. The data are representative of three independent experiments with three biological and two technical replicates. Error bars represent the SD. The number of asterisks adjacent to the symbols, at the same time point, indicates significant differences between different growth conditions (Student’s *t*-test, *p* ≤ 0.05).

To evaluate the effect of the monochromatic light on the cell growth of *E. amylovora* strain Ea273, the microorganism was cultivated in shake flasks under light and temperature-controlled conditions. All cultures were inoculated at the same initial optical density (OD600 = 0.1), with cells from precultures grown in the darkness and in the late exponential phase of growth to minimize the impact of the inoculum on the lag phase of growth. The results reported in Figure 1 indicate that the monochromatic light has had several effects on the growth of this microorganism. Significant differences were observed using OD_600_ as an indirect measure of the cell growth and comparing the OD_600_ values at the end of the growth (on, overnight; 18–24 h after the inoculum). Under the same cultivation conditions (inoculum, temperature, rotation speed, incubation time), the highest cell density (OD_600_ = 4) was achieved for cultivating strain Ea273 under continuous RL (Figure 1). This value was approximately 1.4-fold higher than the one obtained in darkness and 1.2- and 1.3-times higher than the one obtained under FRL and BL. Under BL, the deceleration/decline phase started at least 3 h earlier (8 h after the inoculum), but no significant difference was observed compared to the OD_600_ values at t = 11 and t = on (Figure 1). The latent growth phase was shorter (approximatively 1 h) under FRL compared to darkness (Figure 1). The growth profile under RL showed a prolonged exponential phase (up to t = 8), a prolonged latent phase (up to t = 4), and a higher growth rate in the late phase of growth after the diauxic shift (between 10 and 11 h after the inoculum; Figure 1). These results indicate that monochromatic lighting can modulate the growth pattern of *E. amylovora* independently from the presence of the plant stimuli.

### 2.2. E. amylovora Causes Tissue Necrosis in In Vitro Pear Dar Gazi Plantlets

**Figure 2:**
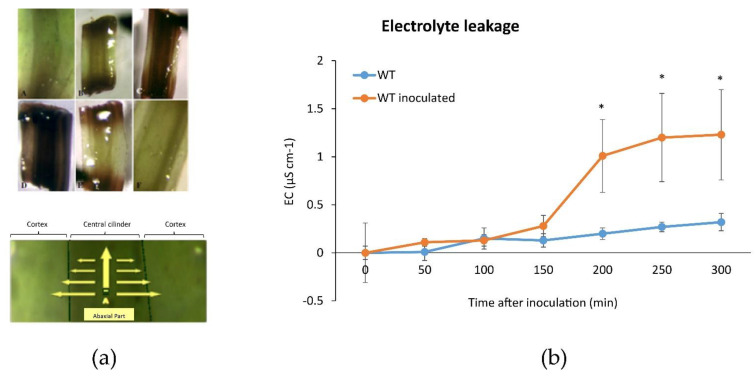
Oxidative activity in inoculated pear plantlets. (**a**) A schematic representation of the oxidative activity in inoculated plantlets with necrotic sections in the stem (upper panel) and necrotic progression from the central cylinder to the cortex, indicated by yellow arrows in the leaf (lower panel); (**b**) Representative data of the electrolyte leakage of inoculated and non-inoculated *Dar Gazi-wt*. Electrolytic conductivity dramatically increased in plants after bacterial inoculation. Error bars represent the SD of three independent experiments, each with three biological replicates. Asterisks indicate significant differences of inoculated vs non-inoculated plants (Student’s *t*-test, *p* ≤ 0.05).

The in vitro grown Iranian pear cultivar Dar Gazi wild type (wt) inoculated with *E. amylovora*, showed oxidative stress in the central cylinder and the cortex of the basal portion of plantlets (Figure 2a). The stems resulted in characteristic signs of HR that cause rapid cell death in the vicinity of the infection point (Figure 2a). These observations are consistent with Electrical Conductivity (EC) measurements, where high values reflect the plasma membrane disruption. An ion leakage three-fold higher was detected in *Dar Gazi-wt* infected vs non-infected (Figure 2b).

### 2.3. Molecular Marker for E. amylovora Infection

**Figure 3:**
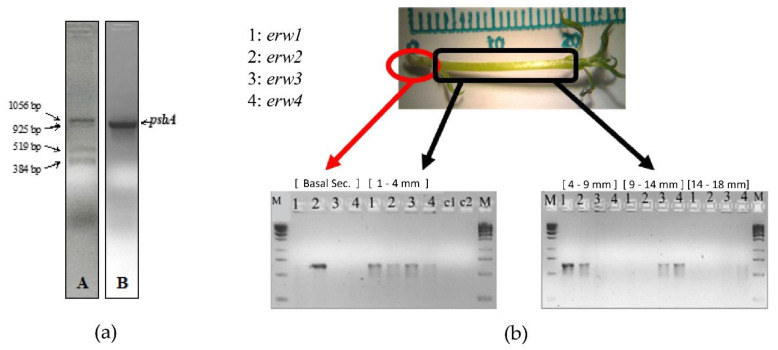
Identification of erw genes: (**a**) Electrophoretic profile of the retrotranscription products (*erw1-4*) obtained from the cDNA synthesized using a psbA gene-specific primer with mRNA from *E. amylovora*-infected plantlets, panel; (**b**) Spatial differential expression in pear tissue of the *E. amylovora* genes revealed using the *erw* genes-specific primers.

A previous study demonstrated that the expression of the chloroplastic gene psbA in the pear cultivar Harrow Sweet is linked to the effects of *E. amylovora* infection [35]. Analysis of psbA expression in inoculated and not-inoculated pear Dar Gazi shoots revealed the presence of unexpected amplicons when we used, as a template, cDNA synthesized with a psbA-specific primer using mRNA extracted from inoculated plants (Figure 3a). The bacterial retrotranscript products were not detected when the cDNA was prepared from non-inoculated shoots or when the cDNA was synthesized using oligo d(T)8-12. Sequencing the PCR products indicated that all of the sequences belonged to *E. amylovora* (sequence identity > 99%). The 1056-bp amplicon, named *erw1*, contains gene sequences encoding: the C-terminal domain of a putative cyclopropane-fatty-acyl-phospholipid synthase (CFAS), an enzyme with synthase and methyltransferase activity involved in the fatty acid biosynthesis; the N-terminal domain of a predicted lipoprotein with an unknown function containing a DUF3833 domain. The 925-bp amplicon (*erw2*) contains the sequence encoding of the predicted Major Facilitator Superfamily (MFS) transporter. These transporters facilitate the transport across cytoplasmic or internal membranes and represent one of the two major classes of transport proteins involved in the protection against endogenous and exogenous toxic compounds in fungi [36]. The 519-bp amplicon (*erw3*) corresponds to the 3′-half of the *erw1* amplicon and contains the sequences encoding the DUF3833 domain-containing protein. The 384-bp amplicon (*erw4*) contains sequences encoding an MFS transporter of the sugar porter (SP) family, the most prominent family of MFS transporter [37]. Gene-specific primers were designed to amplify the same four genes: *erw1*, CFAS; *erw2*, MFS transporter; *erw3*/ *erw1*, DUF3833 protein; erw4, SP MFS transporter. Gene expression analysis was carried out on mRNA extracted from different sections of the asymptomatic pear plantlets (24 h after the infection). The qPCR results indicated that *erw2* was expressed in the basal section up to the middle section (4-9 mm), while *erw1* was expressed in the low- and mid-section (Figure 3b). In contrast, the expression of *erw3* and *erw4* occurred mainly in the mid- and high-section of the plantlet (Figure 3b). This data indicates that, for improving the interaction with the different colonized tissues, *E. amylovora* modulates the expression of its genes during the internal movement through the vascular system.

### 2.4. PR1 and PR10 Expression in Dar Gazi-wt

**Figure 4:**
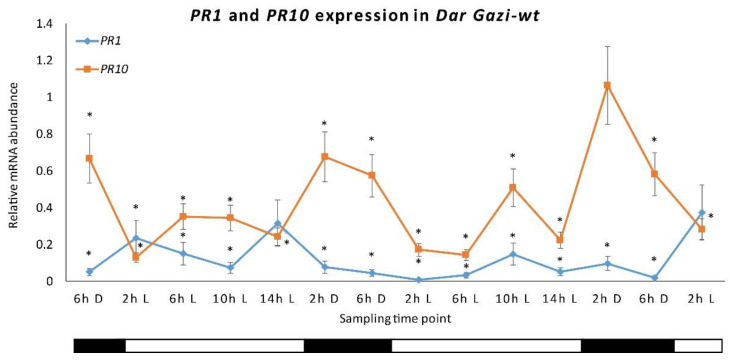
*PR1* and *PR10* expression in *Dar Gazi-wt.* The results are presented after normalization with *ef1A*. The average was generated by two biological replicates run in triplicate. Error bars represent SD. Within the sampling time point, the asterisk indicated a statistically significant difference compared to the highest values of each gene (Student’s *t*-test, *p* ≤ 0.05). The bars under the horizontal axis show the light and dark periods, respectively.

To highlight the role of the internal clock in regulating the in vitro expression of *PR1* and *PR10* genes in Iranian pear cultivar *Dar Gazi-wt*, AtPHYB and LeCRY1 overexpressed lines; plantlets were initially exposed to a photoperiod of 16 h/8 h (light/darkness, Figure S1). In *Dar Gazi-wt*, the expression of the *PR1* gene was not oscillatory, keeping an almost constant level of expressed transcripts throughout the day. The level of *PR1* transcripts was, less than *PR10* transcripts during the day, irrespective of the lighting conditions. *PR10* showed an oscillatory state that would seem to be influenced by the circadian rhythm. The results reported in Figure 4 shows a peak expression after 2 h of exposure to darkness, a tendency to decrease after 6 h of darkness, a strong reduction in the first 2 h of exposure to light and faint up-regulation after 10 h of exposure to light, followed by and a subsequent down-regulation of expression (Figure 4). 

**Figure 5:**
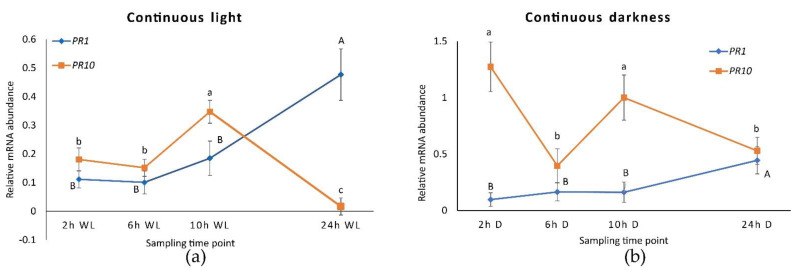
*PR1* and *PR10* expression in *Dar Gazi-wt* plantlets: (**a**) in 24 h continuous light (WL); (**b**) in 24 h continuous darkness. The results are presented after normalization with *ef1A*. Data shown as the average of two biological replicates run in triplicate, with error bars representing SD. For each single gene expression pattern, values with different letters significantly differ according to the analysis of variance (ANOVA) and least significant difference (LSD) tests (*p*  ≤  0.05). Uppercase and lowercase letters are referred to as *PR1* and *PR10*, respectively.

When plantlets were exposed to continuous light (Figure S2), the expression of the *PR1* was approximately doubled after 24 h (Figure 5a) while the expression of the *PR10*, instead, decreased to around zero. This behavior has prevented the peak of expression to be visible after 2 h of exposure to darkness, although a small peak after 10 h of light was observed. Therefore, the expression profile of *PR10* would seem to be independent of the internal clock since the course no longer follows the oscillations previously seen during alternating darkness and light. In fact, in the absence of environmental time cues, circadian rhythms should persist with a period close to 24 h. Under conditions of continuous darkness (Figure 5b and Figure S2), *PR10* expression was stimulated and showed an oscillatory profile that partially resembles what had been observed under photoperiodic conditions. Under continuous darkness, *PR1 r*emains at lower levels than *PR10*, showing the same expression behavior detected during constant light.

### 2.5. PR1 and PR10 Expression in CRY1 and PHYB Overexpressing Lines in WL

**Figure 6:**
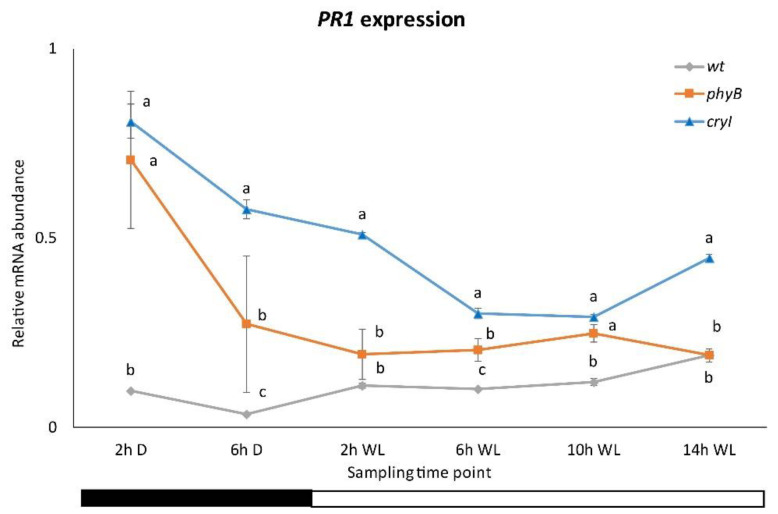
*PR1* expression in *Dar Gazi-wt*, *Dar Gazi-phyB*, and *Dar Gazi-cry1*. Results are presented after normalization with *ef1A*. Data shown as the average of two biological replicates run in triplicate, with error bars representing SD. At each time point, values with different letters significantly differ according to the analysis of variance (ANOVA) and least significant difference (LSD) tests (*p*  ≤  0.05). The bars under the horizontal axis show the light and dark periods.

Data for the *PR1* expression in the plantlets of *Dar Gazi-cry1* line indicate that the photoreceptor CRY1 plays a role in the regulatory system of this gene (Figure 6 and Figure S3). Under darkness, in the plantlets of this line, the detected transcripts increased up to 3 times those detected in the plantlets of Dar Gazy-wt. Moreover, a semi-oscillatory rhythm would seem to be evocated by the increased presence of CRY1 in the plantlet tissues, strongly upregulating the expression of *PR1*. From these results, it was evident that BL plays a role as overexpressed CRY1 emphasizes this aspect. The role of RL turns out to be different than that of BL, as can be seen in the plantlets of the PHYB-overexpressing line (Figure 6). The peak expression of *PR1* during the darkness period was approximately 8-fold greater in the transformed lines relative to the wt-line, comparable to that detected in the plantlets of the *Dar Gazi-cry1*. During the light period, the behavior of gene expression in the plantlets of *Dar Gazi-phyB* is similar to that seen in plantlets of the *Dar Gazi-wt*. During darkness, even in the plantlets of *Dar Gazi-phyB* the *PR1* transcripts level was significantly higher than in the plantlets of *Dar Gazi-wt* (Figure 6).

**Figure 7:**
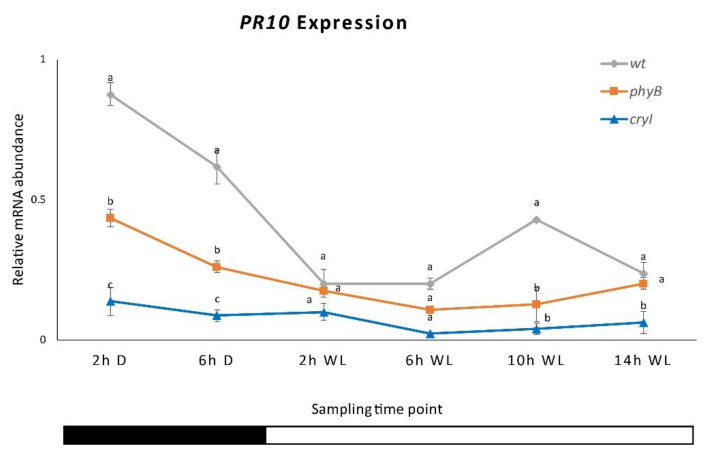
*PR10* expression in *Dar Gazi-wt*, *Dar Gazi-phyB*, and *Dar Gazi-cry1*. Results are presented after normalization with *ef1A*. Data shown as the average of two biological replicates run in triplicate, with error bars representing SD. At each time point, values with different letters significantly differ according to the analysis of variance (ANOVA) and least significant difference (LSD) tests (*p*  ≤  0.05). The bars under the horizontal axis show the light and dark periods.

The analysis of *PR10* gene expression indicated that, in the plantlets of the three *Dar Gazi* lines, the overexpression of each photoreceptor gene drastically reduces the amount of transcript detected (Figure 7 and Figure S3). Furthermore, the oscillatory rhythm detect in the plantlets of *Dar Gazi-wt* results was almost repressed. However, in the overexpressing of PHYB plantlets, expression was maintained in the first 2 h of darkness. 

**Figure 8:**
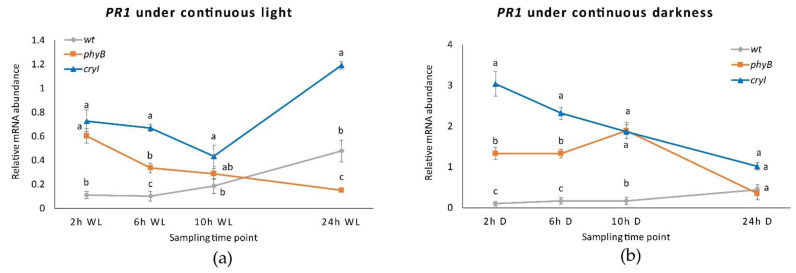
*PR1* expression in *Dar Gazi-wt*, *Dar Gazi-phyB*, and *Dar Gazi-cry1*: (**a**) in 24 h continuous light, panel; (**b**) in 24 h continuous darkness. Results are presented after normalization with *ef1A*. Data shown as the average of two biological replicates run in triplicate, with error bars representing SD. At each time point, values with different letters significantly differ according to the analysis of variance (ANOVA) and least significant difference (LSD) tests (*p*  ≤  0.05).

Under continuous light, although the transcription rate of the *PR1* gene in the plantlets of the *Dar Gazi-cry1* was higher than that in plantlets of the *Dar Gazi-wt*, the behavior of transcription was different than under photoperiodic conditions (Figure 8a). A faint increase in the amount of transcript was detected after 24 h of exposure to continuous light. On the other hand, the *PR1* gene expression course in the plantlets of the *Dar Gazi-phyB*, under exposure to constant light, was similar to that detected under photoperiodic conditions (Figure 8a).

In conditions of continuous darkness, the transcription levels of the *PR1* gene were strongly increased in the tissue of *Dar Gazi-cry1* and *Dar Gazi-phyB*. In contrast, the level of transcript detected in the *Dar Gazi-wt* was very low but did not differ from that seen in continuous light and under photoperiodic conditions (Figure 8b). The highest amount of transcript in the *Dar Gazi-cry1* plantlets was found after 2 h of exposure to the darkness, thereafter, the amount of transcript decreased (Figure 8b). The highest amount of transcript in the plantlets *Dar Gazi-phyB* was found after 10 h of exposure to darkness, but after 24 h, the amount of transcript was the lowest (Figure 8b). Thus, the darkness condition induces always-high *PR1* gene transcription levels in the plantlets of the *Dar Gazi-cry1*. A similar trend was also observed in the plantlet of *Dar Gazi-phyB*, even if at a reduced level.

**Figure 9:**
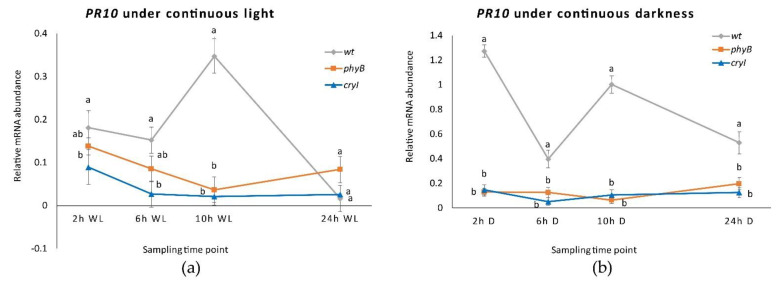
*PR10* expression in *Dar Gazi-wt*, *Dar Gazi-phyB*, and *Dar Gazi-cry1*: (**a**) in 24 h continuous light, panel; (**b**) in 24 h continuous darkness. Results are presented after normalization with *ef1A*. Data shown as the average of two biological replicates run in triplicate, with error bars representing SD. At each time point values with different letters significantly differ according to the analysis of the variance (ANOVA) and least significant difference (LSD) tests (*p*  ≤  0.05).

Under continuous light and darkness, the *PR10* gene expression level was dramatically reduced in plantlets of both transgenic lines (Figure 9a). In the plantlets of the *Dar Gazi-wt*, an oscillatory behavior was detected, more pronounced in continuous darkness than continuous light (Figure 9b). The results suggest that the overexpression of the photoreceptors, irrespective of light conditions, strongly inhibits the expression of the *PR10* gene. The amount of transcript detected in the plantlets of the *Dar Gazi-wt* indicates that the physiological expression of photoreceptors could play a relevant role in permitting the oscillatory expression of the *PR10* gene.

### 2.6. PR1 and PR10 Expression in Dar Gazi-cry1 and Dar Gazi-phyB in RL, FRL, and BL

**Figure 10:**
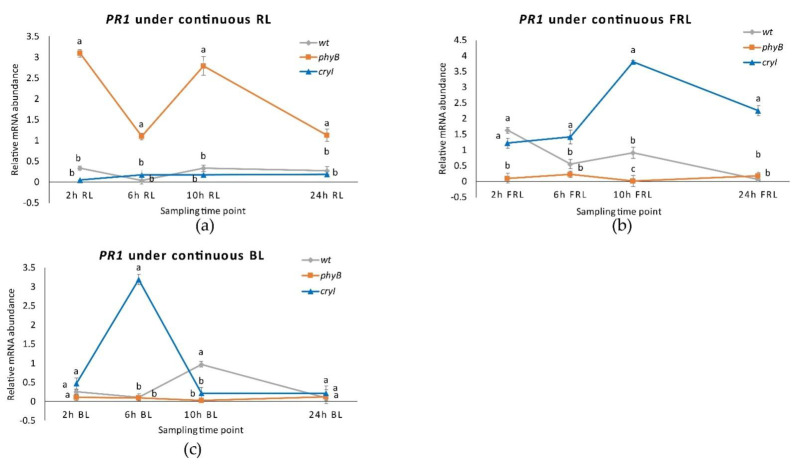
*PR1* expression in plantlets of *Dar Gazi-wt*, *Dar Gazi-phyB*, and *Dar Gazi-cry1*: (**a**) in 24 h continuous RL, panel; (**b**) in 24 h continuous FRL, panel; (**c**) in 24 h continuous BL, panel. Results are presented after normalization with *ef1A*. Data shown are the average of two biological replicates run in triplicate, with error bars representing SD. At each time point values with different letters significantly differ according to the analysis of the variance (ANOVA) and least significant difference (LSD) tests (*p*  ≤  0.05).

Studying the role of photoreceptors in the regulation of the expression of the PRs, phytochrome has a pivotal role in regulating the internal clock and the perception of the photoperiod. The expression level of the *PR1* gene in plantlets *Dar Gazi-phyB* exposed to continuous RL increases to the highest rate (Figure 10a and Figure S3). Moreover, the expression of this gene shows an oscillating trend. On the other hand, in the tissue of *Dar Gazi-wt* and *Dar Gazi-cry1* plantlets, the transcript level was constant, at a very low level of expression. Therefore, the photoconversion of phytochrome from the inactive (P_r_) to the active form (P_fr_) should play a permissive role (Figure 10a,b).

As determined by exposing plantlets to continuous FRL (Figure 10b), the inactive form of phytochrome inhibits the expression of the *PR1* gene in *Dar Gazi-phyB* plantlets. The inactive form of phytochrome, and probably the amount of PHYB protein, either generates or allows an oscillatory behavior of the expression of the *PR1* gene in the plantlets of wt-line and the plantlets of *Dar Gazi-cry1*. The expression of *PR1* in *Dar Gazi-cry1* increases at a high level after 10 h of continuous FRL. Results, therefore, show that *PR1* expression was promoted by CRY1 activity the and the circadian rhythms are present again. 

Under continuous BL conditions (Figure 10c), the highest level of *PR1* expression in *Dar Gazi-cry1* plantlets was reached after 6 h of exposure to light. An oscillatory behavior appeared in plantlets of *Dar Gazi-wt*, while in plantlets of *Dar Gazi-phyB* a very low expression rate without any oscillatory behavior was observed.

**Figure 11:**
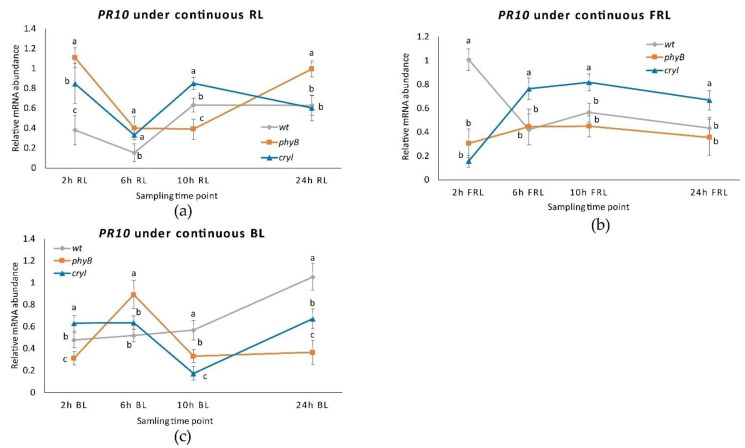
*PR10* expression in *Dar Gazi-wt*, *Dar Gazi-phyB*, and *Dar Gazi-cry1*: (**a**) in 24 h continuous RL, panel; (**b**) in 24 hh continuous FRL, panel; (**c**) in 24 h continuous BL, panel. Results are presented after normalization with *ef1A*. Data shown are the average of two biological replicates run in triplicate, with error bars representing SD. At each time point values with different letters significantly differ according to the analysis of the variance (ANOVA) and least significant difference (LSD) tests (*p*  ≤  0.05).

The regulation of *PR10* expression under continuous RL was very similar in *Dar Gazi* -wt and *Dar Gazi-cry1* plantlets (Figure 11a). An oscillatory transcriptional behavior was observed in both lines of plantlets, although when this behavior is compared to photoperiodic conditions (Figure 11a). In the *Dar Gazi-phyB* plantlets, the trend of oscillatory behavior is different In particular, after the 6th hour, an autonomous behavior was observed (Figure 11a).

When exposed to continuous FRL, the transcriptional profile of *PR10* in plantlets of *Dar Gazi-wt* resemble an oscillatory behavior analogous to that observed in photoperiodic conditions (Figure 11b). This is not the case for the plantlets of the two transgenic lines, that show a different behavior, but are analogous between themselves (Figure 11b). The rate of gene expression observed in *Dar Gazi-cry1* indicates that the presence of CRY1 is required for the upregulation of this gene.

An analogous oscillatory behavior appears in plantlets of both transgenic lines when exposed to continuous BL (Figure 11c), while in *Dar Gazi-wt* not oscillatory behavior was observed. Comparing the rate of the *PR10* gene expression of the plantlets of *Dar Gazi-cry1* under FRL with BL, it is surprising that the behavior was not the same. The hypothesis could eventually explain this divergent behavior, that the gene expression’s promoting role is mainly regulated by phytochrome, and only partly co-regulate by cryptochromes.

### 2.7. CRY1 Overexpressing Line Is More Resistant to Fire Blight

**Figure 12:**
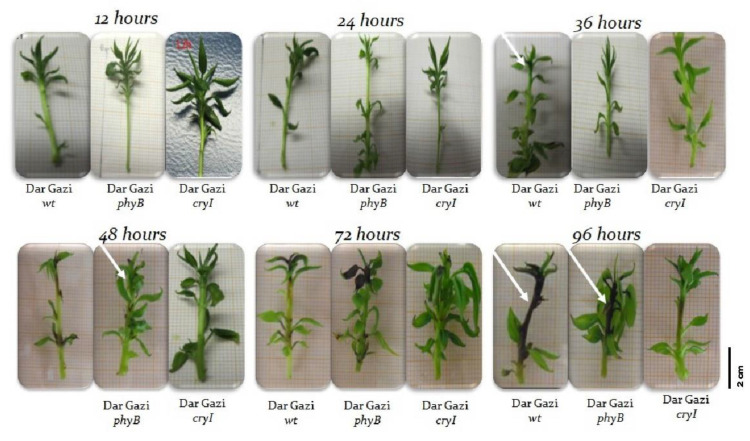
Sintomatology of bacterial infection. White arrows indicate the necrotic stem area.

Plantlets were observed for 96 h after the inoculation of *E. amylovora* to detect necrotic tissues. Necrosis symptoms appeared only in the shoot apex of *Dar Gazi-wt* plantlets after 36 h from the inoculation (Figure 12). After 96 h, the progress of necrosis that affected the entire stem was visible. In the plantlets of *Dar Gazi-phyB*, necrosis was detected in several leaf nodes throughout the stem only after 48 h from the inoculation (Figure 12). Surprisingly, *Dar Gazi-cry1* plantlets better tolerated the pathogen infection showed necrotic tissues after 72/96 h (Figure 12).

**Figure 13:**
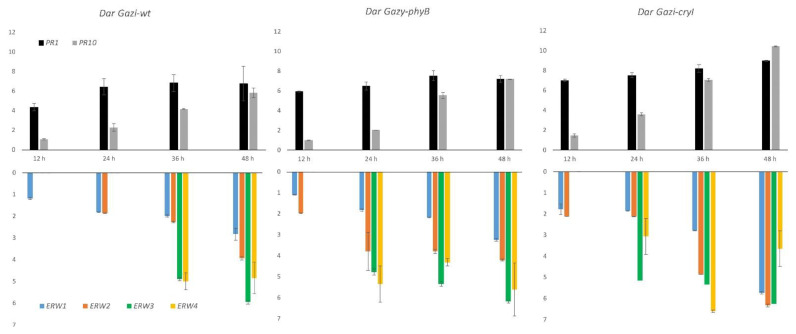
*PR1*, *PR10*, and *erw1*-*4* expression in *Dar Gazi-wt*, *Dar Gazi-phyB*, and *Dar Gazi-cry1* plants, grown under 16/8 h light/darkness, at 12, 24, 36, and 48 h after the pathogen inoculation. Results are presented after normalization with ef1A. Data shown are the average of two biological replicates run in triplicate, with error bars representing SD.

Gene expression analysis showed that transcript levels of PRs were more significant in photoreceptor over-expressing plantlets than in *Dar Gazi-wt* plantlets, indicating an increased capacity to counteract the infection (Figure 13). At the same time, the expression profile of erw genes indicated that only *erw1*, coding for CAFS, was expressed during the first 12 h after the infection. In addition, erws transcript levels were also greater in plantlets over-expressing photoreceptors than in *Dar Gazi-wt* plantlets, suggesting a dynamic interaction occurring during the bacterial invasion of host tissues (Figure 13). 

## 3. Discussion

Plants have evolved to coordinate their activities with the day-night cycle by Earth’s rotation. Direct responses to light and darkness are essential, but, in addition, biological clocks have evolved to time biological processes. Circadian rhythms result from the interaction between the internal oscillatory system and the receptors of environmental cues such as photoreceptors that usually help reset the biological clock to a 24-h day-night cycle. Many environmental (i.e., temperature) and internal cues (i.e., starvation) function as zeitgebers for the rhythms, but photoperiod and light quality are among the most important ones in plants. There is no other environmental factor in any climatic region of comparable importance for the immediate control of annual and daily cycles [38].

### 3.1. Regulation of PR Genes by Circadian Rhythms and Photoreceptors

This research shows that the expression of the *PR10* gene is partially regulated by the internal biological clock, while photoreceptors mainly control the *PR1* gene. The expression of the *PR10* gene in plantlets exposed to continuous RL and FRL under a 16/8 h photoperiod (Figure 7) maintained an oscillatory pattern, which appeared to be controlled by the circadian rhythm (Figure 11a,b). In contrast, the transcript of the *PR1* gene appeared to be independent of the oscillator and dependent on the photoreceptor’s activities. These agree with the observations by Genoud et al. [5] on the effect of single and multiple nil mutants in the light perception (*phyA* and *phyB*) and the light-signal processing (*psi2*, phytochrome signaling) on the interaction between *A. thaliana* and the pathogen *Pseudomonas syringae* pv. *tomato* single and multiple mutants’ nil in light perception (*PHYA* and *PHYB*) and light-signal processing (*psi2*, phytochrome signaling). 

In these mutants, the growth of an incompatible bacterial strain of this pathogen was enhanced in the double mutant *phyAphyB* and decreased in the *psi2* mutant under darkness and dim light conditions [20]. The last mutant increased the light signal transduction regulated by PHYA and PHYB [39]. Similarly, the results of this work demonstrated that the overexpression of *PHYB* and *CRY*1 is associated with an upregulation of the Dar Gazi *PR1* (Figure 6 and Figure 8). 

Salicylic acid (SA) induces pathogen-related gene expression and accumulation of related proteins, and its production also depends on the light regime [40]. Phytochromes are required for the expression of the PR1 protein [19]. In *Dar Gazi-phyB*, the expression of *PR1* was dependent on the phytochrome. When the plantlets were exposed to continuous RL an up-regulation of *PR1* was observed (Figure 10a); in contrast, in plantlets exposed to continuous FRL, the expression of this gene was inhibited (Figure 10b). When the plantlets were exposed to FRL and BL, a co-participation of the cryptochrome into the regulation system was also observed (Figure 10b,c).

Although in this study, free SA was not measured, it is known that the SA levels oscillate throughout the day in a circadian rhythm [41], so a fine coordinated regulation of *PR1* gene between this hormone and photoreceptors pathways could be strongly hypothesized, and it will be the challenge for the further investigation. It has been shown that transcription of *PR* genes during plant defense involves a key transcriptional regulator of SA signaling known as Nonexpressor of Pathogenesis-related protein 1 (NPR1). The inactive NPR1 oligomers monomerize in the cytosol after an SA-induced change of the cell’s redox state, and a circadian oscillation occurs, peaking at night [42]. The state of monomers allows NPR1 to be translocated to the nucleus where they interact with TIMING OF CAB2 EXPRESSION 1 (TOC1), an evening circadian clock gene, and TGACG-BINDING FACTORs (TGAs), leading to the expression of defense-related genes involved in the set-up of plant immune defense, including *PR* genes [42,43,44,45]. The oscillatory rhythms of TOC1 mRNA expression were associated with parallel oscillations in histone acetylation [46]. NPR1 forms an activator complex with histone acetyltransferases (HATs) HAT1 and HAT5. Through NPR1–TGA interaction, the complex is recruited to chromatin finally relaxing genomic DNA and facilitating *PR*s transcription activation [47].

The obtained data suggest that the *PR*s clock-associated regulation is co-regulated by the photoreceptors phytochrome and cryptochrome, maybe functionally as elements of regulator-Zeitlupe systems. In fact, in overexpressing *PHYB* gene plantlets, a circadian oscillation is observed when exposed to a continuous RL (Figure 9 and Figure 10). The results indicate that this behavior is red/far-red reversible. When plantlets are exposed to FRL and BL, a co-participation of cryptochrome into the regulation system was also observed.

In overexpressing *phyA* cherry plants, Cirvilleri et al. [48] concluded that the induction of *PR*s gene is strictly dependent on light quantity and quality, inducing plant resistance to *Pseudomonas syringae* pv. *mors-prunorum*. Therefore, the relationship between biological clock and overexpression of *PHYB* and *CRY1* was tested under different light qualities to unravel their role on *PR*s gene expression pear cv Dar Gazi. 

In a previous study, a possible link between *PR1* and light was postulated [40]. However, until now there has been a gap of knowledge around the role of the internal clock in the *PR1* regulome. The effects of many biotic and abiotic stress, including pathogen infection, salt tolerance, UV irradiation, and ozone stress, have been investigated in *PR10* gene expression [49]. These stresses have been shown to activate *PR10* gene expression, suggesting their importance during plant defense responses. Plant hormones and related signaling molecules have been reported to regulate *PR10* gene expression, including jasmonic acid, salicylic acid, abscisic acid [50], kinetin, and auxin [51]. 

### 3.2. Light Plays a Role in E. amylovora Growth

Under standard laboratory conditions, a non-photosynthetic micro-organism is grown in the darkness, and the possible effects of light on its growth and physiology are neglected. This practice is strongly consolidated, and, for this reason, microbial culture equipment (static and shaken incubators) is not provided with a light control system in the standard configuration. In contrast, there are several studies on non-photosynthetic bacteria associated with humans, plants, and animals (i.e., *Pseudomonas aeruginosa*, *Pseudomonas syringae,* and *Xanthomonas*) indicating that chemical (quorum sensing), and light (photosensing) signals affect the growth pattern, infectivity, and virulence of these bacteria through common regulatory pathways [30,52] Data presented in this work provide the first evidence that the spectral distribution of the light affects the growth of *E. amylovora* under laboratory conditions. This preliminary result provides novel prospects in studying the impact of spectral quality on the lifestyle of this phytopathogen and its interactions with the plant host.

### 3.3. Cryptochrome Increase the Defense against the Attack of E. amylovora

In this paper, four *E. amylovora* genes were identified that can be used to monitor the diffusion of the pathogen in pear vascular tissues during asymptomatic and symptomatic periods (Figure 3). Quantitative expression analyses revealed several interesting features: the expression of the *erw* genes was modulated during the internal movement of the pathogen through the plant vascular system (Figure 3); the activation of these genes occurred at specific times during the infection, the temporal expression pattern was dependent upon the pear genotype (Figure 13). In both *Dar Gazi* transgenic lines, the expression of *erw2*, *erw3* and *erw4* was advanced by 12 h compared to *Dar Gazi-wt*, from 24 h to 12 h for *erw2*; 36 h to 24 h for *erw3* and *erw4* (Figure 13). Twelve h after the infection, the transcript levels of *erw1* in *Dar Gazi-wt* and *Dar Gazi-phyB* were similar and increased in the same proportion between 12 h and 24 h (Figure 13). These data suggested that, in infected *Dar Gazi-wt* and *Dar Gazi-phyB* tissues, the growth pattern and the number of *E. amylovora* cells per plant mass unit were comparable. The early activation of *erw2* and the increased *erw2*/*erw1* ratio at 24 h in *Dar Gazi-phyB* vs. *Dar Gazi-wt* were dependent on the overexpression of *PHYB* in the transgenic line (Figure 13).

In contrast, 12 h after the infection, the mRNA expression level of *erw1* in the transgenic *Dar Gazi-cry1* line was about two-fold higher than in *Dar Gazi-wt*, and this difference remained constant up to 48 h (Figure 13). These data indicated that the overexpression of *CRY1* stimulated *E. amylovora* growth in pear tissues and altered the expression of the other erw genes. Noteworthy, in *Dar Gazi-phyB*, the expression of *erw2* and *erw4* remained constant between 12 h and 48 h (Figure 13). At 48 h, there was no significant difference in the expression levels of the four *erw* genes between *Dar Gazi-wt* and *Dar Gazi-phyB* (Figure 13). In contrast, in the *Dar Gazi-cry1* line, the transcript levels of *erw1*, *erw2*, and *erw3* significantly increased between 36 h and 48 h, reaching the maximum relative abundance. In comparison, the expression of *erw4* had a maximum at 36 h and decreased between 36 h and 48 h (Figure 13). These data indicated that the plant-pathogen interactions occurring during the pathogen invasion were differentially affected by the alterations of the phytochrome- and cryptochrome-modulated signals resulting from the overexpression of *PHYB* and *CRY1*.

In the pear tissue, pathogen invasion generated oxidative stress in the central cylinder and the cortex accompanied by a widespread disruption of the plasma membrane that developed from the basal portion to the apex of the plantlets (Figure 2a). In *Dar Gazi-wt*, the necrosis symptoms appeared 12–36 h earlier than in transgenic lines (Figure 12).

Interestingly, in the plantlets over-expressing the photoreceptors, the transcript levels of *PR1* and *PR10* were higher than in *Dar Gazi-wt* (Figure 13). Independently from the pathogen load estimated by the *erw* genes expression data, it should be noted that there was a correlation between the PR transcript level and the appearance of necrosis symptoms. Delayed symptoms occurred in the *Dar Gazi* lines, such as *Dar Gazi-cry1* (Figure 12), in which higher *PR1* and *PR10* transcription levels were observed (Figure 13). It was demonstrated that a wide range of endogenous and exogenous (a)biotic factors, including pathogen attack, accumulation of salicylic acid, and abiotic stress, can regulate temporally and spatially the expression of *PR* genes [53,54] and the secretion and accumulation of the corresponding proteins in the apoplastic space or the vacuoles [55]. The results of this work demonstrate that the accumulation of the PR proteins can interfere with the dynamic interaction occurring during the *E. amylovora* invasion and delay the infection of the pear host tissues.

It is known that the protein product of the Far-red Insensitive 219/Jasmonate Resistant1 (FIN219/JAR1) functions as a jasmonic acid (JA)-conjugating enzyme responsible for the synthesis of the Jasmonic Acid-isoleucine (JA-Ile), the physiologically active form [56]. Under BL, FIN219 plays a role in the regulation of phenotype development and bacterial resistance [57,58] and how it occurs under FRL, it interacts with CONSTITUTIVE PHOTOMORPHOGENIC 1 (COP1), down-regulating also the levels of *COP1* and up-regulating the levels of *HY5* [59]. COP1 is involved in the negative control of nitrate reductase activity in Arabidopsis *cop1* mutant, reducing the availability of nitrogen [60]. The availability of nitrogen resulted responsible for both *Arabidopsis* resistance to *E. amylovora*, i.e., under nitrogen limitation, the resistance decreased due to the lower apoplastic reactive oxygen species (ROS) accumulation and increased expression of *E. amylovora hrps* genes [61,62]. Moreover, cryptochromes may work together with phytochromes to modulate plant defense responses. In *Arabidopsis*, CRY1 positively regulates the inducible resistance to *P. syringae* pv. *tomato*. The local resistance is down-regulated in the *cry1* mutant; in contrast, in plants overexpressing *CRY1*, the *PR1* gene expression is enhanced, and the resistance is significantly up-regulated [22]. These results agree with the increased expression level observed in *Dar Gazi-cry1* compared to in *Dar Gazi-wt*, where a significant increase of expression was already detected at 12 h from inoculation, for both *PR1* and *PR10* genes.

Although, many key molecular factors involved in the plant-pathogen interaction, from the plant perception of the pathogen (P/MAMPs, PRRs) to the activation of the PAMP-triggered immunity (PTI), and the Effector triggered immunity (ETI), are already known [63,64], in *E. amylovora*-infected plants, the regulation of the photoreceptors by the interaction with the major phytohormones, SA, JA, and ethylene remains to be explored.

### 3.4. Agronomic Relevance

In a fruit orchard, the canopy dimension dynamically changes, and, consequently, the spectral distribution of the incoming radiation varies widely, as the light penetrates and scatters within the tree canopy due to the structure and optical properties of plant organs [65,66]. In general, the spectral modifications of light inside the tree canopy have a crucial role in growth partitioning among fruit and shoots, affecting the allocation to developing fruits in plant growth and fruit quality [67]. The effects of modification of the CRYs and PHYs abundance and photosensitivity of plants in response to the changing light on cross talks during host-pathogen interaction remain to be studied in fruit trees, and the molecular mechanisms underlying the interaction of monochromatic light with plant and bacteria remain poorly understood because they are influenced by environmental conditions. Results obtained in experiments in vitro, with pure cultures of *E. amylovora* Ea273 strain (Figure 1), and in vivo, with infected transgenic *Dar Gazi* lines (Figure 12), clearly indicated that the quality of the light and the photoreceptor-mediated signals affect the growth of the pathogen and its infectivity and aggressiveness. In this respect, the use of the LED technology can be valuable to develop new procedures for sustainable and non-invasive control of this pathogen.

These findings also have great economic importance because *PR1* is used as a lookout pathogen presence. During the period of fruit conservation in dark conditions, an interruption of these light conditions through BL flesh could repress the insurgence, the development, and bacterial proliferation. Even if there are not many studies on *PR*s and woody fruit crop plants, it has recently been presented that genetically engineered phytochrome A cherry plants showed the highest level of tolerance to *Pseudomonas syringe* pv *mors-prunorum*, when compared to the wild type plants [48].

Finally, one of the four plant food allergens, the Bet v 1 superfamily, contains ten pathogenesis-related proteins [68]. Our findings could be further explored to study the regulation of this allergen-related protein and the relative reduction of its presence and accumulation by modulating the lighting during the post-harvest fruit conservation.

## 4. Materials and Methods

### 4.1. Plant Material, Medium Composition, Growth Conditions, and Bacterial Strain

An in vitro-cultured plantlets system of *Pyrus communis* L. cv Dar Gazi was used to evaluate if the internal clock autonomously regulates the abundance of *PR1* and *PR10* transcripts. Plantlets of three different lines: *Dar Gazi-wt*, *Dar Gazi-phyB,* and *Dar Gazi-cry1* were submitted to different circadian experimental conditions and continuous BL, RL- and FRL conditions. Fluorescent WL was used as a control. 

The two transgenic lines *Dar Gazi-phyB* and *Dar Gazi-cry1* were obtained starting from leaf explants of in vitro established cv *Dar Gazi-wt* co-cultivated for 20 min on MS liquid basal medium with two different *A. tumefaciens* strains (A, B) prepared as described below.

The disarmed *A. tumefaciens* (A) strain EHA 105, contained the helper plasmid pTiBo542 and the binary vector pROKB (kindly provided by Whitelam, Leicester University, England), harboring the *neomycin phosphotransferase II* (*nptII*) gene under the control of *nos* promoter and the *A. thaliana* cDNA *PHYB* gene under the control of the cauliflower mosaic virus 35S RNA (CaMV 35S) promoter. The disarmed *A. tumefaciens* (B) strain EHA 105, contained the helper plasmid pTiBo542 and the binary vector pBI12 (also provided by Whitelam), harboring the *neomycin phosphotransferase II* (*nptII*) gene under the control of *nos* promoter and *Lycopersicum esculentum* cDNA *CRY1* gene under the control of the CaMV 35S promoter. Vectors were introduced into EHA 105 using freeze-thaw transformation of *Agrobacterium* and *Escherichia coli* as described by [69]. For both transformation experiments *A. tumefaciens*, was cultured overnight at 28 °C on a shaker at 80 rpm in 10 mL liquid Luria-Bertani (LB) [70] medium, prepared with 1% LB containing Bacto-Tryptone, 0.5% Bacto-yeast extract, 1% NaCl, 0.1% glucose with 100 mg L^−1^ of kanamycin added when the temperature arrived at 45° C for selecting bacteria carrying the binary plasmid. After 10 min of centrifugation at 3200× *g*, the pellet was resuspended in MS liquid medium with 3% (*w*/*v*) sucrose, and subsequent dilutions were done to reach a final concentration of around 0.3 (OD600).

After the co-cultivation, the leaf explants were transferred to a regeneration medium containing 100 mg L^–1^ acetosyringone (40-hydroxy-3,5-dimethoxyacetophenone) and incubated in a controlled environment chamber at 23 °C for two days. Cefotaxime (200 mg L^−1^) was added to all media, to eliminate *Agrobacterium*. The transformed green shoots were picked out from callus tissue (assisted by a stereoscope) and moved to QL0 medium [71] with 10 mg L^−1^ of kanamycin added (Figure S4a). The final selection was carried out onto QL0 medium added with 100 mg L^−1^ of kanamycin, subculturing every 8–10 days to select putative transgenic lines (Figure S4b). 

### 4.2. Molecular Confirmation of the Transgene Insertion

The selected shoots were subcultured four/five times for rapid and clonal multiplication onto the PQL1 media contained the same composition present in PQL0 and enriched with 0.7 mg L^−1^ BAP.

Based on the assumption that genes encoding for the same proteins in different species show conserved domain with a high degree of identity, divergent regions between pear *PHYB* gene and *AtPHYB*, and between pear *CRY1* and *LeCRY1* were selected to design specific primers. The selected regions were checked against bacteria genes as well and they did not match any homologous eukaryotic sequences of genes present in data banks. *AtPHYB* and *LeCRY1* sequences, expected product sizes and annealing temperatures used to detect each gene are presented in Table S1.

PCR amplification tests were conducted to test to validate the insertion of both genes on plantlets of selected lines (Figure S4c). For DNA extraction procedure, from 100 mg leafy shoot tissues, and PCRs chemicals and amplification profile have been using the procedure reported in previous work [72]. Amplification products were visualized on agarose gels (1.2%, *w*/*v*) and 10 µg mL^−1^ of ethidium bromide (Figures S6 and S7).

### 4.3. E. amylovora In Vitro Experiments

*E. amylovora* strain Ea273 was obtained from American Type Culture Collection (ATCC number 49946). The strain was stocked at −80 °C in LB plus glycerol 25% (*v*/*v*) and precultured in LB broth at 30 °C under agitation (150 rpm) in the absence of light. The growth was monitored by turbidimetric measurements (OD_600_). 

Seed cultures in the late exponential phase of growth [OD_600_ of 4.5–4.8] were used to inoculate 100 mL of LB medium (initial OD_600_ of 0.1), to evaluate the effect of the light quality on the growth. The inoculated broth was grown in 500-mL Erlenmeyer flasks in an INFORS HT Multitron incubator equipped with monochromatic LED lights of the appropriate spectral wavelength. The growth was carried out under constant temperature (30 °C) and agitation (180 rpm), in continuous light or darkness, and was monitored over a 24-h period. All experiments were carried out in triplicate and included three biological and two technical replicates.

### 4.4. Growth Conditions and Sampling

*PR1* and *PR10* expression of each line, *Dar Gazi-wt*, *Dar Gazi-phyB,* and *Dar Gazi-cry1*, was evaluated in plants grown in vitro according to Abdollahi et al. (2004) [2] at 22 °C and under a 40 μmol m^−2^ s^−1^ WL. For the light experiments, plants were then placed under different light conditions: WL (100 µmol m^−2^s^−1^), RL (25 µmol m^−2^ s^−1^), FRL (25 µmol m^−2^s^−1^) or BL (25 µmol m^−2^ s^−1^) obtained using specific LED lamps. Light quality and quantity were measured with an EPP 2000 Fiber Optic Spectrometer (StellarNet Inc., Tampa, Florida, USA). Plants were harvested as reported in supplemented Figures S1–S3. 

### 4.5. RNA Isolation and Quantification

A pool of plants of the three different lines was ground with mortar and pestle in liquid nitrogen. According to the manufacturer’s instructions, two independent total RNA extractions were performed from each pool using the kit NucleoSpin RNA plant (Macherey-Nagel), according to the manufacturer’s instructions. Total RNA was treated using Invitrogen™ TURBO DNA-free™ Kit (Thermo Fisher Scientific, Milano, Italy) to remove DNA contamination. The nucleic acid purity was analyzed by Thermo Scientific™ NanoDrop™ 2000/2000c Spectrophotometer (Thermo Fisher Scientific) and samples with 260/280 and 260/230 nm absorbance ratios greater than 1.8 nm were used for the following experiments.

### 4.6. cDNA Synthesis and qRT-PCR

According to the manufacturer’s instructions, total RNA was retro-transcripted employing gene-specific primer and random primers of Invitrogen™ SuperScript™ II Reverse Transcriptase (Thermo Fisher Scientific). cDNA was used as a template for qRT-PCR reactions. The PCR reactions were performed in technical triplicates with the LightCycler 480 SYBR Green I Master reagent using the LightCycler^®^ 480 Instrument (Roche, Italy) in 96-well reaction plates. PCR conditions were: one cycle at 95 °C for 5 min, followed by 40 cycles of 95 °C for 15 s and 60 °C for 30 s. At the end of the PCR, to confirm the presence of a unique amplicon, the melting curve was evaluated and a single peak in every reaction was observed. Relative template abundance was quantified using the standard curve method [73] and the *Elongation Factor 1-Alpha* (Accession: AY338249.1) was used as a reference gene for expression normalization. PCR efficiency was estimated using six-point, 10-fold, diluted standard curves. Means from two independent replicates were subjected to SD calculation and Student’s t-test. The primers were designed using the Primer3 software web version 4.1.0 (Table 1).

### 4.7. Ion Leakage Assay

*Dar Gazi-wt* infected with *E. amylovora* were collected 24 h after the inoculation and cut at the basal side which was submerged in the media. Plants were washed in de-ionized water twice and placed in 25 mL of de-ionized water. Each time point had triplicate samples for infected and non-infected plants. Solution conductivity was measured using a handheld conductivity meter, Type RS 180-7127 (RS Components), at the indicated times after plant collection.

## 5. Conclusions

The main findings of this study shed light on the role of light quality and reveal a possible mechanistic control of photoreceptors on the signaling transduction that activates the plant genetic resources to respond to the *E. amylovora* pathogen attack via a large array of transcription factors. CRY1 has an agonistic role in the activation of *PR* genes, during the interaction of host-pathogen, and an antagonistic role in the *E. amylovora* growth. These results suggest that a possible escape signal joined to circadian and ultradian rhythms could be connected to the regulome of PR proteins synthesis under BL and their photosensor, which might play a relevant role in plants grown in an orchard. Moreover, the results provide new knowledge on fire blight control methods targeting the plant light regulation systems. The sensitivity of the *E. amylovora* to monochromatic radiation could use LED technology for sustainable and non-invasive pathogen control. New scenarios in plant pathology control systems through th77e defense gene activation by light and negative regulation of pathogen virulence could be operational in the frame of optogenetic control [74].

To our knowledge, no previous study has addressed the effect of the environmental spectral quality’s radiation constraints on the activation of the genes involved in plant-pathogen interaction. It remains to be found whether or not SA, JA, and ethylene genes play a role in the blue-light signaling (COP1, HYH, SPA1) and if they have a regulatory relationship with CRY1. Future studies could focus on the use of light quality, in particular BL, as an elicitor to set up protection methods for fruit crop trees, in the nursery and orchard, against fire blight.

## Figures and Tables

**Table 1:** The sequences of primers of PRs pear genes and erw genes, used in the qRT-PCR reactions.

Primer Name	Forward	Reverse	Amplicon Size (bp)
*ef1A*	GTTCGAGAAGGAGGCTGCTGAG	CGAACTTCCACAGGGCAATGTCA	119
*PR1*	CTCGAGCAGCAGTAGGCGTTG	CATGTTGGTTGGCGTAGTTTTGT	180
*PR10*	AGGAGACATTGAAATTAAGGAAGAA	AGTTGTATGCGTCGGGGTGGT	167
*erw1*	GCGATTACCATCAGCGAAGAAC	CCCATCTCAAACTGGTCAACAAC	161
*erw2*	GCTGGTGCTTGCTGTTGTTTC	GGACGCTTTCAGTTCGTGTGT	103
*erw3*	CTGTTACTGACGCTTTGCCTGT	CCGCTGTAATCCTGTACCATCC	140
*erw4*	ACCCTGTTCGTCTGTTTCCTTG	CGATCCACTCTTGTTGATGAGG	130

## Data Availability

The data presented in this study are available on request from the corresponding author.

## Supplementary Materials

The following are available online at https://www.mdpi.com/article/10.3390/plants10091886/s1, Figure S1: Representative scheme of the sampling time point used to evaluate the PR genes expression in Dar Gazi-wt grown under white light, Figure S2: Representative scheme of the sampling time point used to evaluate the *PR* genes expression in *Dar Gazi-wt* exposed for 24 h under continuous lightness (a) and continuous darkness (b), Figure S3: Representative scheme of the sampling time point used to evaluate the *PR* genes expression in *Dar Gazi-wt*, *Dar Gazi-phyB* and *Dar Gazi-cry1* plants grown in exposed for 24 h under continuous WL, RL, FRL or BL, Figure S4: Dar Gazi events of regenerations after 25 days in dark condition (a). Shoots were transferred to the medium, containing kanamycin, to select putative transgenic lines, and subcultured four/five times for rapid and clonal multiplication (b). The medium used for the in vitro selection of regenerated buds was enriched with 100 mg/L Kanamycin. (c) Plantlets of transgenic lines during proliferation state, Figure S5: Detection of *ef1A* gene fragments in cDNA of *Dar Gazi-wt*, *Dar Gazi-phyB,* and *Dar Gazi-cry1* plants (a). M: Ladder; WT: *Dar Gazi-wt*; phyB*: Dar Gazi-phyB*; cryI: *Dar Gazi-cry1*; B: Blank. Detection of *NptII* gene fragments in cDNA of *Dar Gazi-wt*, *Dar Gazi-phyB,* and *Dar Gazi-cry1* plants (b). M: Ladder; WT: *Dar Gazi-wt*; phyB: *Dar Gazi-phyB*; cryI: *Dar Gazi-cry1*; B: Blank, Figure S6: Detection of *AtPHYB*, using specific primers for *A. thaliana PHYB* that amplify the *AtPHYB* gene fragments only in cDNA of *Dar Gazi-phyB* plants (a). M: Ladder; WT: *Dar Gazi-wt*; phyB: *Dar Gazi-phyB*; cryI: *Dar Gazi-cry1*; B: Blank. Detection of *LeCRYI*, using specific primers for *L. esculentum CRYI* that amplify the cryILE gene fragments in cDNA of *Dar Gazi-cry1* plants (b). M: Ladder; WT: *Dar Gazi-wt*; phyB: *Dar Gazi-phyB*; cryI: *Dar Gazi-cry1*; B: Blank, Table S1: Sequence of primers used to validate the molecular insertion of *AtPHYB* and *LeCRY1* in pear genome.
