# Supplementary material for: Monochromic Radiations Provided by Light Emitted Diode (LED) Modulate Infection and Defense Response to Fire Blight in Pear Trees
Source: Plants (Basel). 2021 Sep 12;10(9):1886. doi: 10.3390/plants10091886 (PMC8465259; doi:10.3390/plants10091886)
Supplement: Supplementary file 1 [file plants-10-01886-s001.zip › plants-1372807-supplementary.pdf]

MONOCHROMIC RADIATIONS PROVIDED BY LIGHTEMITTED DIODE (LED) MODULATE INFECTION AND DEFENSE RESPONSE TO FIRE BLIGHT IN PEAR TREES

Tiziana Sgamma [1,†], Ivano Forgione [2,†], Francesca Luziatelli [3], Calogero Iacona [4], Roberto Mancinelli [2], Brian Thomas [5], Maurizio Ruzzi [3], and Rosario Muleo [2,*]

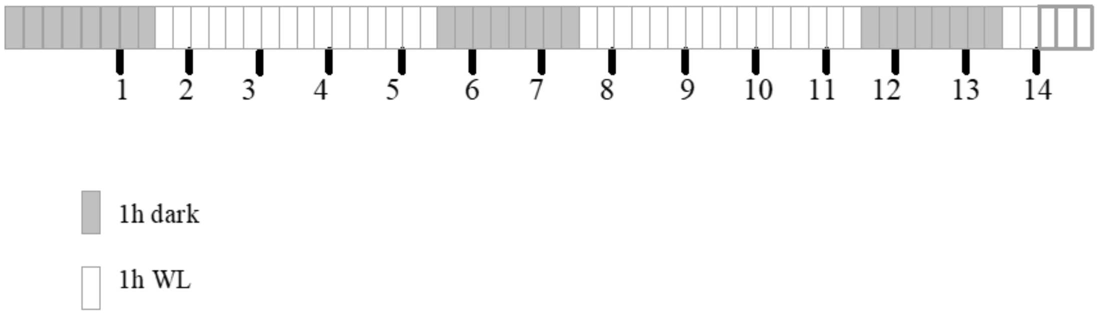

Samples	
1	6h dark
2	2h light
3	6h light
4	10h light
5	14h light
6	2h dark
7	6h dark
8	2h light
9	6h light
10	10h light
11	14h light
12	2h dark
13	6h dark
14	2h light

Figure S1. Representative scheme of the sampling time point used to evaluate the *PR* genes expression in *Dar Gazi-wt* grown under white light.

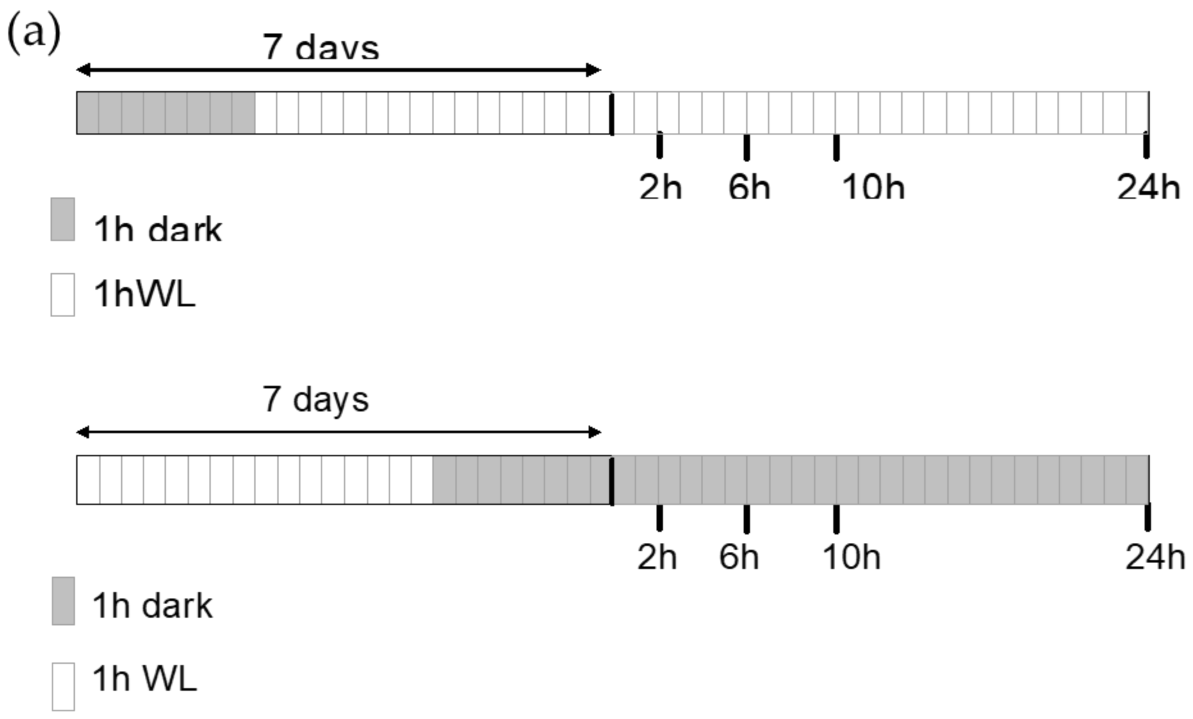

Figure S2. Representative scheme of the sampling time point used to evaluate the *PR* genes expression in *Dar Gazi-wt* exposed for 24 h under continuous lightness (a) and continuous darkness (b).

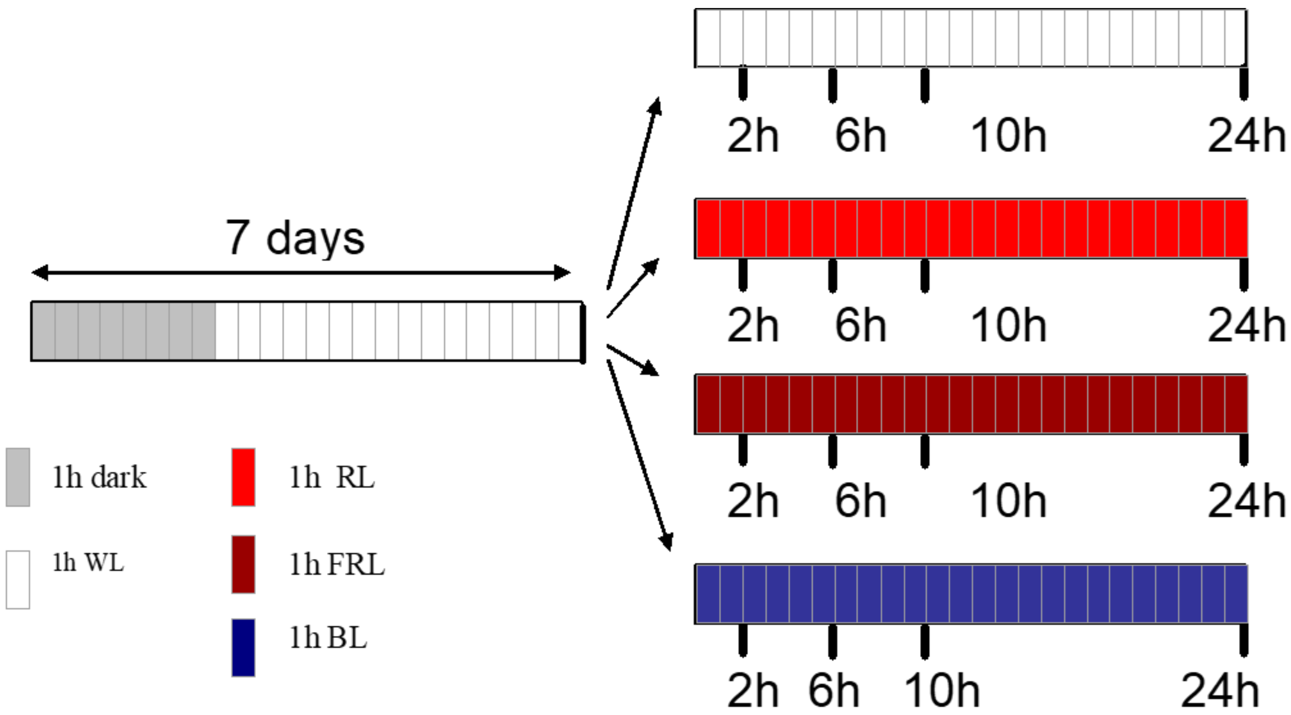

Figure S3. Representative scheme of the sampling time point used to evaluate the *PR* genes expression in *Dar Gazi*-wt, *Dar Gazi-phyB* and *Dar Gazi-cry1* plants grown in exposed for 24 h under continuous WL, RL, FRL or BL.

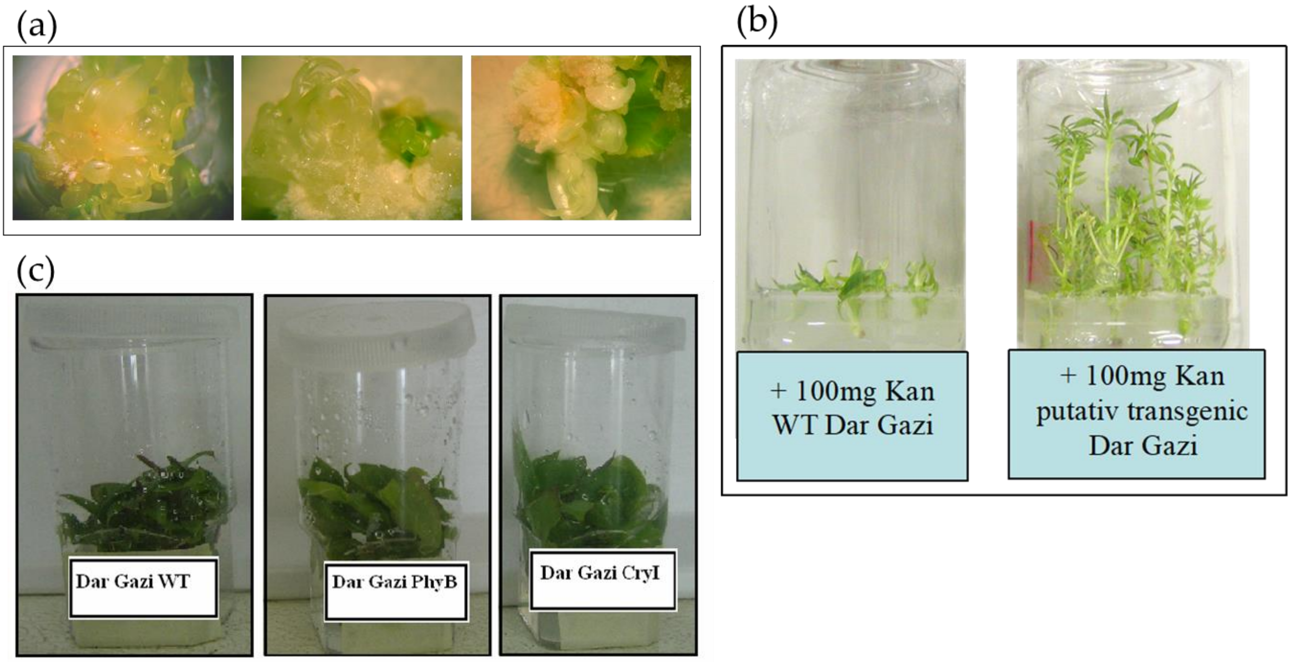

Figure S4. Dar Gazi events of regenerations after 25 days in dark condition (**a**). Shoots were transferred to medium, containing kanamycin, to select putative transgenic lines, and subcultured four/five times for rapid and clonal multiplication (**b**). The medium used for the in vitro selection of regenerated buds was enriched with 100 mg/l Kanamycin. (**c**) Plantlets of transgenic lines during proliferation state.

**Gene**	**Gene Product**	**Primer Forward**	**Primer Reverse**	**T a**	**Amplicon Size (bp)**
phyB	*Arabidopsis thaliana* pbyB	catgaagatgagcatggagaag	cgagcttcttccactagctac	59	345
cry1	*Lycopersicon esculentum* cry1	gcttcctgaacttgctaggc	ggttgttcctgactggttca	55	263
nptII	*neomycin phosphotransferase II*	atggattgcacgcaggttct	ccaacgctatgtcctgatagc	58	658

Table S1. Sequence of primers used to validate the molecular insertion of *AtPHYB* and *LeCRY1* in pear genome.

Among the transgenic events two were chosen, named phyB and cry1, for the photobiological modified behaviour and tested with further PCR analysis and used with *Dar Gazi* wild type for the experiments described in this work. In Figure S6 the amplicons obtained.

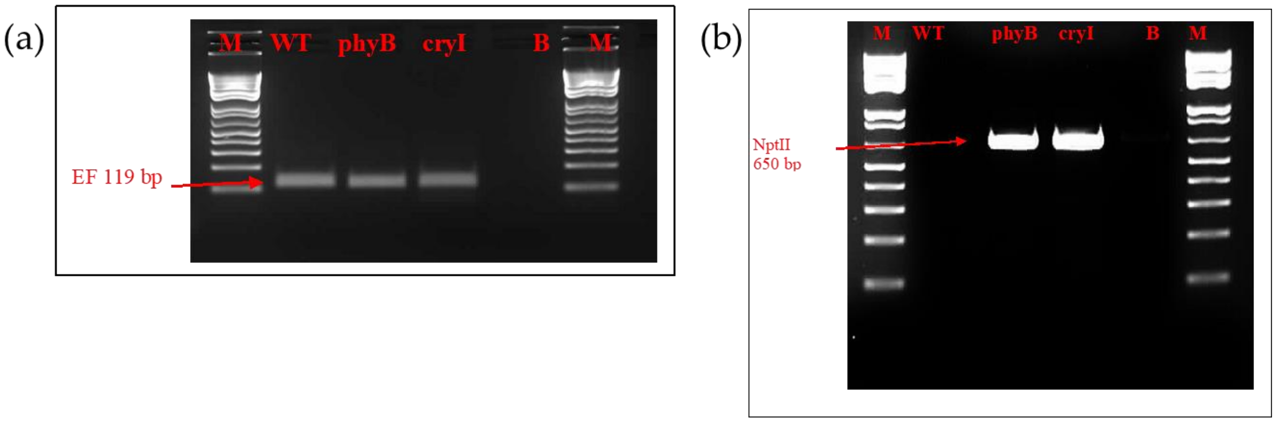

Figure S5. Detection of *ef1A* gene fragments in cDNA of *Dar Gazi-wt*, *Dar Gazi-phyB* and *Dar Gazi-cry1* plants (**a**). M: Ladder; WT: *Dar Gazi-wt*; phyB: *Dar Gazi-phyB*; cryI: *Dar Gazi-cry1*; B: Blank. Detection of NptII gene fragments in cDNA of *Dar Gazi-wt*, *Dar Gazi-phyB* and *Dar Gazi-cry1* plants (**b**). M: Ladder; WT: *Dar Gazi-wt*; phyB: *Dar Gazi-phyB*; cryI: *Dar Gazi-cry1*; B: Blank.

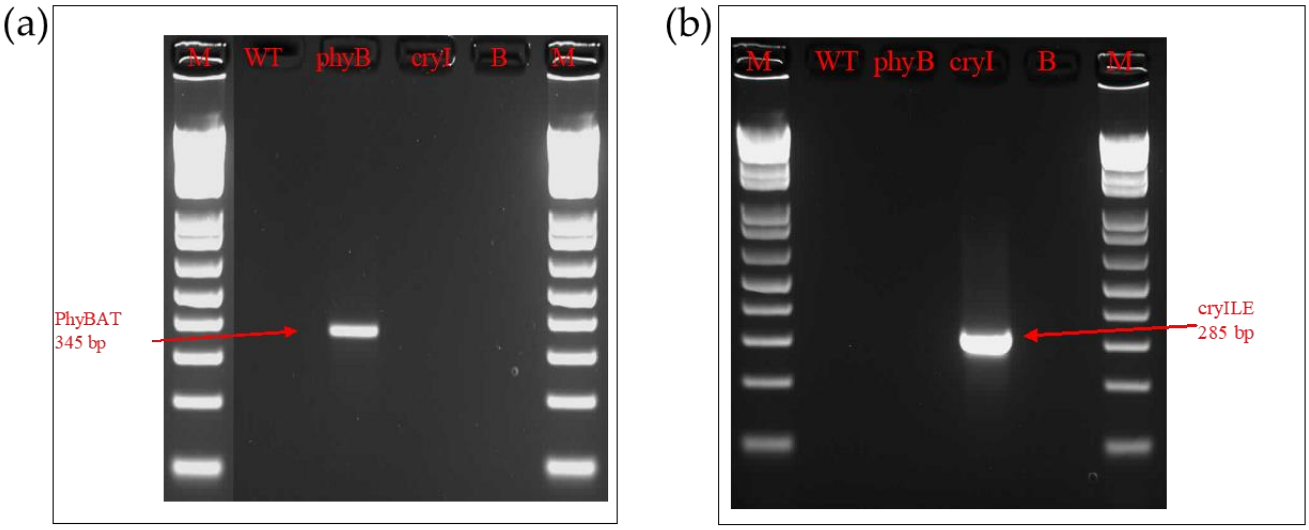

Figure S6. Detection of *AtPHY*B, using specific primers for *Arabidopsis thaliana PHYB* that amplify the PhyBAT gene fragments only in cDNA of *Dar Gazi-phyB* plants (**a**). M: Ladder; WT: *Dar Gazi-wt*; phyB: *Dar Gazi-phyB*; cryI: *Dar Gazi-cry1*; B: Blank. Detection of *LeCRYI*, using specific primers for *Lycopersicon esculentum CRYI* that amplify the *cry*ILE gene fragments in cDNA of *Dar Gazi-cry1* plants (**b**). M: Ladder; WT: *Dar Gazi-wt*; phyB: *Dar Gazi-phyB*; cryI: *Dar Gazi-cry1*; B: Blank.
